# First-Generation Cephalosporins for Treatment of Acute Hematogenous Osteomyelitis in Children: A Study of Efficacy and Adverse Effects

**DOI:** 10.1093/ofid/ofad610

**Published:** 2023-12-14

**Authors:** Lisa Hiskey, Hiba Saifuddin, Emily R Levy, Roland Hentz, Nipunie S Rajapakse, Laura M Dinnes, Elizabeth H Ristagno

**Affiliations:** Division of Pediatric Infectious Diseases, Department of Pediatric and Adolescent Medicine, Mayo Clinic, Rochester, Minnesota, USA; Division of Plastic and Reconstructive Surgery, Department of Surgery, Louisiana State University, New Orleans, Louisiana, USA; Division of Pediatric Infectious Diseases, Department of Pediatric and Adolescent Medicine, Mayo Clinic, Rochester, Minnesota, USA; Division of Pediatric Critical Care Medicine, Department of Pediatric and Adolescent Medicine, Mayo Clinic, Rochester, Minnesota, USA; Division of Clinical Trials and Biostatistics, Department of Quantitative Health Sciences, Mayo Clinic, Rochester, Minnesota, USA; Division of Pediatric Infectious Diseases, Department of Pediatric and Adolescent Medicine, Mayo Clinic, Rochester, Minnesota, USA; Department of Pharmacy, Mayo Clinic, Rochester, Minnesota, USA; Division of Pediatric Infectious Diseases, Department of Pediatric and Adolescent Medicine, Mayo Clinic, Rochester, Minnesota, USA

**Keywords:** cefadroxil, cephalexin, OPAT, osteomyelitis, pediatric

## Abstract

**Background:**

Acute hematogenous osteomyelitis (AHO) is a relatively infrequent but significant infection in pediatric patients. As *Staphylococcus aureus* is the most common cause of AHO, intravenous and oral first-generation cephalosporins are common therapies. Cephalexin is a commonly prescribed oral therapy for pediatric AHO, although it requires frequent dosing that may affect adherence. Cefadroxil is a comparable oral first-generation cephalosporin with a more desirable dosing schedule.

**Methods:**

We reviewed pediatric patients admitted to Mayo Clinic between March 2002 and September 2020 for management of AHO who received treatment with a first-generation cephalosporin. We reviewed timing of oral therapy transition, therapy-associated adverse effects, and recurrence of disease after completion of therapy.

**Results:**

There were 59 patients included in the study. There was similar occurrence of adverse effects in patients receiving cefadroxil and cephalexin, although use of cefadroxil coincided with more gastrointestinal adverse effects and leukopenia and use of cephalexin with more rash and neutropenia. One secondary treatment failure occurred in our study, in a patient receiving cephalexin for treatment of septic arthritis.

**Conclusions:**

Cefadroxil may be a reasonable alternative oral therapy for methicillin-susceptible *S aureus* or culture-negative AHO in pediatric patients, particularly when a less frequent dosing schedule is desired. Future study with a larger sample size is warranted.

Acute hematogenous osteomyelitis (AHO) is a bacterial infection of the bone that occurs as a result of the hematogenous spread of bacteria [1]. This condition primarily affects young children and occurs in approximately 8 per 100 000 children per year in high-income countries [1, 2]. The most common bacterial cause of osteomyelitis is *Staphylococcus aureus*, with methicillin-susceptible *S aureus* (MSSA) and methicillin-resistant *S aureus* (MRSA) implicated [3]. Other bacterial causes include *Streptococcus pyogenes*, *Streptococcus pneumoniae*, and *Kingella kingae* [1, 4–6]. Additionally, it is common for cases to yield negative culture results [1, 7, 8]. Empiric therapy is aimed at treating the most common causes of osteomyelitis, considering a patient's age and risk factors, and antibiotic therapy is later adjusted in concordance with available culture results [1].

Because the most common cause of AHO is *S aureus*, empiric antimicrobial therapy for patients without risk factors for other organisms (eg, hemoglobinopathies) is aimed at *S aureus*. Common empiric antibiotics include cefazolin, oxacillin, or nafcillin. Empiric therapy should include coverage of community-acquired MRSA in cases of high local prevalence (>10%–20%) or patient risk factors (eg, known colonization, previous infection) [2].

Initial treatment is commonly given via the intravenous (IV) route, with subsequent transition to oral or outpatient parental antibiotic therapy (OPAT) to complete treatment. The decision to transition to oral therapy or OPAT is based on each patient's specific clinical scenario and response to treatment [2]. In uncomplicated cases, oral therapy has been shown to result in similar clinical courses and rates of relapse as prolonged IV therapy [9, 10]. In contrast, OPAT has been associated with increased medication-related adverse effects as well as central venous catheter complications resulting in increased emergency department visits, additional anesthetic encounters, and readmissions [9, 11, 12]. Because of this, transition to oral therapy is recommended over OPAT in children for whom an appropriate oral antibiotic is available and tolerable [2].

When transition is made to oral therapy, infections proven to be caused by MSSA and culture-negative AHO in areas with low community prevalence of MRSA are commonly treated with oral cephalexin, a first-generation cephalosporin. Due to its half-life of approximately 1 to 3 hours, cephalexin is typically administered every 6 to 8 hours [13]. One concern with using cephalexin is that this higher frequency of dosing potentially reduces adherence [14, 15]. Cefadroxil is another first-generation cephalosporin with the same antimicrobial spectrum of coverage and a similar adverse effect profile [16, 17]. However, cefadroxil has the benefit of a slightly longer half-life, allowing for less frequent dosing of every 8 to 12 hours [16–19]. One recently published single-center experience showed successful treatment of musculoskeletal infections with cefadroxil (30 mg/kg/d divided twice daily) [20]. Despite these recent data, overall research into the use of cefadroxil in the treatment of pediatric AHO is lacking—so much so that cefadroxil is excluded from the Pediatric Infectious Diseases Society/Infectious Diseases Society of America clinical practice guideline for diagnosis and management of AHO in pediatrics [2].

We conducted a single-center retrospective cohort study including all cases of AHO in children <18 years of age between March 2002 and September 2020 at the Mayo Clinic. We compared adverse effects and clinical outcomes in children who completed antibiotic therapy with either cephalexin or cefadroxil.

## METHODS

This was a single-center retrospective cohort study. Pediatric patients (<18 years of age) treated at Mayo Clinic in Rochester, Minnesota, between March 2002 and September 2020 were eligible for inclusion in the study. Patients were identified via a provider-compiled database of infectious disease consults during the study period. This database was compiled by infectious disease physicians entering patient and diagnosis information into the database at the time of consultation, for the purpose of recording these consultations for later review, research, and education. Patients with MSSA or culture-negative AHO with or without concurrent septic arthritis who received cefadroxil or cephalexin for oral therapy were included. Patients were excluded if infection was associated with trauma or surgical hardware or if the infection was caused by an organism other than MSSA (as proven by culture). All included patients had consented to use of Minnesota medical records for research purposes. Data were collected via chart review from the electronic medical record. Patient charts were reviewed from hospital admission, through discharge, and until completion of treatment with 1 of the 2 antibiotics. Patients were diagnosed with treatment failure if they developed a sustained increase in white blood cells or inflammatory markers (C-reactive protein, sedimentation rate), new fever without alternative cause, or indication for further surgical intervention for management of osteomyelitis. Primary treatment failure was defined as a failure to respond to initial therapy; secondary treatment failure was defined as an initial good clinical response with later recrudescence during therapy. Recurrence was defined as new clinical symptoms of osteomyelitis within 6 months of completion of therapy [2].

Summary statistics compared cefadroxil and cephalexin. Analysis was conducted with SAS and R programming languages [21, 22]. Outcome variables were clinically reported side effects, abnormal laboratory values, and treatment failure. Only adverse events that occurred during the treatment period for a specific drug were attributed to that drug.

Laboratory test results were considered abnormal based on cutoffs of the continuous laboratory value appropriate to each patient's age and sex. For total white blood cell count, absolute neutrophil count, and erythrocyte sedimentation rate, Mayo Clinic laboratory reference values were used. Total eosinophil count was considered abnormal if >0.5 eosinophils/L and C-reactive protein ≥8 mg/L. Age-based reference ranges for alanine aminotransferase and creatinine were obtained from the *Harriet Lane Handbook* [23]. Alanine aminotransferase was considered elevated if ≥5 times the upper limit for age. Creatinine was considered elevated if ≥1.5 times the upper limit for age. A patient was considered to have a persistently abnormal laboratory value if there were 2 consecutive abnormal results for that test within a 2-week period.

The association between the oral antibiotic group and the outcome variables was tested with the Fisher exact test. The exact 95% CIs were included. Odds ratios (ORs) are presented to compare cefadroxil and cephalexin. Days to oral antibiotic transition for patients receiving cefadroxil and cephalexin were summarized with a Kaplan-Meier plot. Differences between groups were tested with the log-rank test.

Missing data were limited but most prevalent for laboratory tests. There was no observed outcome for a laboratory result if the patient had no tests of that type. Patients with a single laboratory test with a normal value had an observed outcome of no abnormal value.

## RESULTS

There were 59 patients included in the study. Of these, 30 received cefazolin followed by cefadroxil, and 29 received cefazolin followed by cephalexin. Patient demographics are presented in Table 1. The median age was 10 years. There was comparable sex, race, and ethnicity demographics across all groups. Most patients (66%) had osteomyelitis of a long bone; 2 had spinal osteomyelitis; and the remainder of patients had osteomyelitis of another location. Nine patients had concurrent septic arthritis. Two patients in the cephalexin group underwent 2 procedures for management of AHO during initial admission; the remainder underwent 1 or no procedures for management. Thirty-one patients had at least 1 blood or operative culture positive for MSSA; 28 patients had no positive cultures. One patient had 1 positive blood culture for *Micrococcus* that grew after 38 hours; this was deemed a contaminant by the treating clinical team.

**Table 1.** Demographics of Pediatric Patients in the Study

	Cefadroxil (n = 30)	Cephalexin (n = 29)	Total (n = 59)
Age, y			
Mean (SD)	11 (5)	7 (4)	9 (5)
Median (IQR)	12 (8–14)	7 (3–11)	10 (4–13)
Sex			
Male	16 (53)	15 (52)	31 (53)
Female	14 (47)	14 (48)	28 (47)
Race			
White	29 (97)	24 (92)	53 (95)
Other	1 (3)	2 (8)	3 (5)
Missing data	0	3	3
Ethnicity			
Hispanic or Latino	1 (3)	2 (8)	3 (5)
Not Hispanic or Latino	29 (97)	23 (92)	52 (95)
Missing data	0	4	4

Data are presented as No. (%) unless noted otherwise.

**Figure 1.**
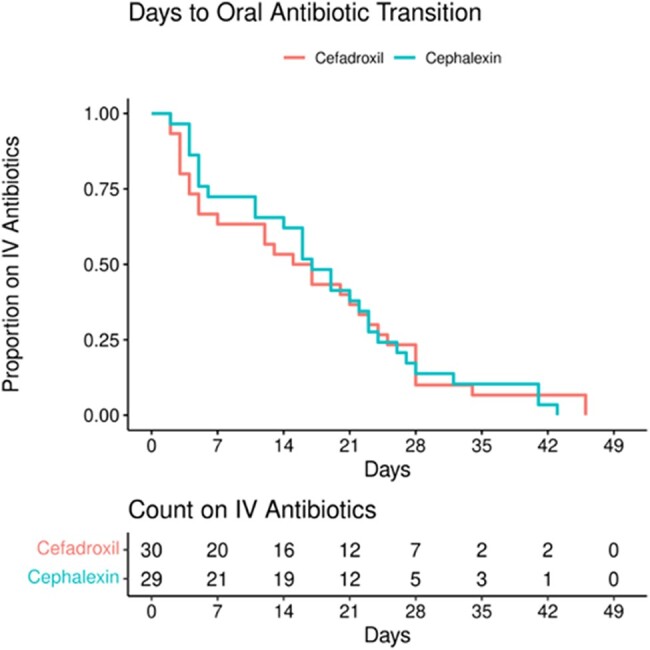
Days to oral antibiotic transition. In the graph, the blue line represents patients who received oral therapy with cephalexin, and the red line represents patients who received oral therapy with cefadroxil. The median time for transition to oral therapy was 17 days for transition to cephalexin, and 16 days for transition to cefadroxil. The chart presents the number of patients receiving IV antibiotic treatment at the noted points in therapy. Nineteen patients received ≤7 days of IV therapy prior to transition to oral treatment; 8 of these patients transitioned to cephalexin and 11 to cefadroxil. A log-rank test comparing the 2 groups did not find evidence of a difference in timing of transition to oral therapy (*P* = .94).

The median daily dose for cephalexin was 88 mg/kg/d (IQR, 63–100) with most patients (n = 18, 62%) receiving cephalexin divided every 6 hours. The median dose for cefadroxil was 51 mg/kg/d (IQR, 38–59), and all patients received cefadroxil divided every 12 hours. Timing for transition from cefazolin to oral therapy is illustrated in Figure 1. The median time for transition to oral therapy was 16 days for cefazolin to cefadroxil and 17 days for cefazolin to cephalexin. Nineteen patients received ≤7 days of IV therapy prior to transition to oral treatment; 8 of these patients transitioned to cephalexin and 11 to cefadroxil. A log-rank test comparing the 2 groups did not find evidence of a difference in timing of transition to oral therapy (*P* = .94). There was a noted trend toward shorter IV courses over time, consistent with general trends in pediatric infectious disease practice. Over the course of the study period, median days of IV therapy decreased from 25 days between 2002 and 2007 to 18 days between 2008 and 2013 and 11.5 days between 2014 and 2019.

Adverse effect frequencies are presented in Table 2. There were no statistically significant differences between groups. Rash was slightly more common in patients receiving cephalexin (n = 2, 7%) vs cefadroxil (n = 0, 0%; OR, 0 [95% CI, 0–3.3]; *P* = .24). Gastrointestinal side effects (n = 2 [7%] vs n = 0 [0%]; OR, infinity [95% CI, .28–infinity]; *P* = .49) and leukopenia (n = 1 [5%] vs n = 0 [0%]; OR, infinity [95% CI, .045–infinity]; *P* > .99) were slightly more common with cefadroxil vs cephalexin. Both occurrences of gastrointestinal side effects with cefadroxil occurred in patients who received ≤7 days of IV therapy. The remainder of patients who experienced adverse effects while taking cephalexin or cefadroxil received >7 days of IV antibiotic therapy. Patients who developed adverse effects with either medication received similar dosing to those who did not develop adverse effects.

**Table 2.** Adverse Effects Between the Groups

	Medication Administered When Adverse Effects Occurred, No. (%)			
Adverse Effect	Cefadroxil (n = 30)	Cephalexin (n = 29)	OR (95% CI)	*P* Value
Nausea, vomiting, and/or diarrhea	2 (7)	0 (0)	Infinity (.28–infinity)	.49
Rash	0 (0)	2 (7)	0 (0–3.3)	.24
Leukopenia	1 (5)	0 (0)	Infinity (.045–infinity)	>.99
Missing data	9	11	…	…
Neutropenia	0 (0)	1 (6)	0 (0–15)	.44
Missing data	10	13	…	…
Eosinophilia	0 (0)	0 (0)	…	…
Missing data	11	17	…	…
Elevated liver enzymes	0 (0)	0 (0)	…	…
Elevated creatinine	0 (0)	0 (0)	…	…

Abbreviation: OR, odds ratio.

One patient experienced secondary treatment failure. This patient developed worsening evidence of osteomyelitis following treatment for septic arthritis with initially questionable associated bony abnormalities. This patient was diagnosed with MSSA left hip septic arthritis and underwent irrigation and debridement twice while hospitalized. The patient received 7 days of IV antibiotic therapy (ceftriaxone and vancomycin, followed by cefazolin) and was discharged taking cephalexin (100 mg/kg/d divided every 6 hours). The patient clinically improved but did have a persistent mild limp, intermittent pain, and persistently elevated erythrocyte sedimentation rate with normalization of C-reactive protein. The patient had completed nearly 4 weeks of therapy with cephalexin when he developed a rise in inflammatory markers as well as magnetic resonance imaging findings suggestive of osteomyelitis of the left femoral head. He completed an additional 3 weeks of cefazolin followed by 1 additional week of cephalexin with no further recurrence. There were no cases of treatment failure in patients who received oral therapy with cefadroxil. There were no cases of recurrence or chronic osteomyelitis in either group within 6 months of completion of treatment.

## DISCUSSION

Our single-center retrospective study demonstrates similar clinical outcomes in patients receiving cefadroxil (median dose, 51 mg/kg/d divided every 12 hours, up to 2000 mg/d) vs cephalexin (median dose, 88 mg/kg/d divided every 6 or 8 hours, up to 4000 mg/d). One patient in the cephalexin group experienced secondary treatment failure with rise in inflammatory markers and change in magnetic resonance imaging findings while receiving treatment for MSSA septic arthritis of the left hip. No patients in the cefadroxil group experienced treatment failure, and there was no recurrence among patients in either group within 6 months of completion of therapy. Medication adverse effects were similar in number between groups, though varied in nature: gastrointestinal symptoms and leukopenia occurred in more patients receiving cefadroxil, while rash and neutropenia occurred in more patients receiving cephalexin.

Other studies have demonstrated success in use of cefadroxil for treatment of AHO in children [19]. Previous recommendations have suggested cefadroxil dosing of 100 to 150 mg/kg/d divided 2 or 3 times per day, for treatment of osteomyelitis, though more recent literature has demonstrated success with much lower doses (30 mg/kg/d divided twice per day) [20, 24]. Our study adds to growing data regarding successful use of cefadroxil in pediatric patients, particularly at lower doses as compared with previous recommendations. Additionally, our study contributes a comparison of outcomes and adverse effects in patients receiving cefadroxil and cephalexin for AHO within the same period. Based on recent results combined with our data, cefadroxil at lower doses (30 to 60 mg/kg/d divided twice per day) may be a reasonable alternative to cephalexin for oral transition therapy in patients with MSSA or culture-negative AHO.

Limitations of this study include small sample sizes in all groups and low frequencies of measured outcomes. Due to these limitations, estimates were based on few observations of each outcome, and statistical measures of uncertainty were large. It is also notable that 68% of patients received ≥7 days of IV cefazolin, which may decrease the likelihood of finding a meaningful difference between oral therapy groups. Some clinical information and laboratory data were limited due to the electronic medical record transition during the study period. Additional data that may have been helpful in comparing groups were not available for all patients, including duration of fever or bacteremia. Furthermore, the database used to collect patient information was compiled by manual provider entry, with the potential for unintentionally missing qualifying patients during our study period.

In the future, additional study (eg, cohort study) comparing cephalexin and cefadroxil for treatment of AHO in children may be helpful to better elucidate the use and optimal dosing of cefadroxil in these patients.

## Notes

***Author contributions.*** L. H., E. H. R., N. R., E. R. L., and L. D. contributed to study conception and design. L. H. and H. S. contributed to data collection. R. H. contributed to data analysis. L. H. prepared the manuscript. All authors reviewed the results, contributed manuscript edits, and approved the final version.

***Data access statement.*** Due to the sensitive nature of the research, supporting data are not available.

***Patient consent statement.*** All participants gave informed consent for use of Minnesota medical records for research purposes. Approval to conduct research was obtained from the institutional review board at Mayo Clinic, Rochester, Minnesota, USA.
